# RPS2: a novel therapeutic target in prostate cancer

**DOI:** 10.1186/1756-9966-28-6

**Published:** 2009-01-12

**Authors:** Min Wang, Youji Hu, Mark E Stearns

**Affiliations:** 1 Department of Pathology, Drexel University College of Medicine, 15th and Vine Streets, MS435 NCB1, Philadelphia, PA 19102-1192, USA

## Abstract

**Background:**

A number of studies have previously shown that the over expression of different ribosomal proteins might play an important role in cancer (i.e. S3a, L10, L16). We have previously reported that RPS2, a 33 Kda ribosomal protein was over expressed in malignant prostate cancer cell lines and in archived tumor specimens. Thus, RPS2 or other aberrantly over-expressed ribosomal proteins might promote cancer and be excellent therapeutic targets for treatment of the disease.

**Methods:**

Western blotting and RT-PCR have been used to measure and compare the levels of expression of RPS2 in a variety of malignant prostate cancer cell lines, plus normal and benign cells lines. We have developed a 'ribozyme-like' DNAZYM-1P '10–23' motif oligonucleotide and examined whether it targets RPS2 in different cell lines by RT-PCR and Western blots. Growth and apoptosis assays were carried out to measure whether DNAZYM-1P 'knock-down' of RPS2 influenced cell proliferation or survival. We have also developed a SCID mouse tumor model with PC-3ML cells to determine whether DNAZYM-1P targeting of RPS2 compromised tumor growth and mouse survival rates *in vivo*.

**Results:**

Western blots showed that PC-3ML, LNCaP, CPTX-1532, and pBABE-cmyc stably transfected IBC-10a cells all over-expressed RPS2, whereas IBC-10a parent, NPTX-1532, and BPH-1 cells or mouse NIH-3T3 cells expressed barely detectable levels of RPS2. RT-PCR assays showed that DNAZYM-1P, which targeted RPS2, 'knocked-down' RPS2 expression in the malignant cells (i.e. PC-3ML cells) *in vitro*. The DNAZYM-1P also inhibited cell growth and induced apoptosis in the malignant prostate cells, but had little effect on the normal IBC-10a or NPTX-1532 cell lines. Finally, SCID mouse tumor modeling studies showed that DNAZYM-1P blocked tumor growth and metastasis by PC-3ML cells and eventually eradicated tumors following localized or systemic i.v. delivery. Mouse survival studies revealed that there was a dosage dependent increase in disease free survival rates in mice treated systemically with DNAZYM-1P (i.e. mouse survival increased from 0% to 100%).

**Conclusion:**

In sum, we have shown for the first time that therapeutic targeting of RPS2 is an excellent approach for the eradication of prostate cancer in preclinical tumor modeling studies.

## Background

Although our understanding of their role in cancer is limited, the expression of a variety of ribosomal proteins has been associated with the development of prostate and colon cancer. For example, we have previously reported that RPS2, a 33 Kda ribosomal protein was over expressed in malignant prostate cancer cell lines and in archived tumor specimens [1]. Vaarala et al. [2] found that L7a and L37 ribosomal proteins were over-expressed in prostate-cancer cell lines and in prostate cancer tissue samples. Furthermore, L23a- and S14-transcript levels were significantly elevated in PC-3 cells as compared to a normal prostate epithelial cell line termed PrEC [2]. Utilizing 'micro-quantity differential display', Bee et al. [3] found L19 (RPL19) was 5-fold higher in malignant prostate cell lines and 8-fold higher in malignant tissues, when compared with their benign counterparts of human prostate [3]. The authors suggested that expression of RPL19 protein could be a valuable marker in prostate cancer diagnosis and patient management. Similarly, Pogue-Geile et al. [4] found that the RPS3, RPS6, RPS8, RPS12, RPL5, and PO ribosomal proteins were expressed at higher levels in 8 different colon adenocarcinomas and adenomatous polyps. These results suggest that a select pool of ribosomal proteins might be elevated in prostate and colon cancer during the transformation process and play a key role in tumorigenesis.

Previously, we have cloned a mutant variant of the RPS2 ribosomal protein, termed PCADM-1, and shown that RPS2 was over expressed in malignant prostate cell lines and in human prostate cancer (PCa) [1]. PCADM-1 was over-expressed in human PCa and not found in benign (BPH), high grade prostatic intraepithelial neoplasia (HGPIN), or seminal vesicle (SV) tissue. Likewise, the normal RPS2 gene was found to be over-expressed by malignant prostate lines (i.e. PC-3 ML and LNCaP cells), and by early stage prostate cancer cell lines (HGPIN, CPTX-1532). The data suggest that PCADM-1 and/or RPS2 might be novel bio-markers and excellent prognostic indicators for human prostate cancer. More importantly, PCADM-1 or RPS2 might be novel therapeutic targets for treating the disease.

In this paper, we have examined the importance of the RPS2 gene for proliferation and survival of malignant and normal prostate cell lines *in vitro *and *in vivo*. We have developed a 'ribozyme-like' oligonucleotide, DNAZYM-1P, which specifically targets RPS2 and found that DNAZYM-1P treatment of PC-3ML, LNCaP, and CPTX-1532 cells induced a significant increase in cellular apoptosis and death (i.e. > 95% after 48 hr). Mouse tumor modeling studies further revealed that DNAZYM-1P delivered locally or systemically, eradicated primary and metastatic tumors of PC-3ML cells in SCID mice. More importantly, treatment dramatically increased mice disease free survival rates by 100%. For the first time, we have convincingly demonstrated that tumors which over express the RPS2 protein can be eradicated with a DNAZYM-1P targeting this gene.

## Methods

### Cell cultures

LNCaP, DU145, CRW22R1 and mouse 3T3 fibroblasts were obtained from ATCC (Bethesda, MD) and grown according to their instructions. PC-3 ML cells were maintained in DMEM plus 10% fetal bovine serum according to published methods [5]. CPTX-1532 and NPTX-1532 cells were derived from malignant and normal tissue of the same human prostate tissue, respectively [6]. BPH-1 cells [7] were a gift from Donna Peehl (Stanford Univ.). CPTX-1532, NPTX-1532, and BPH-1 cells were each immortalized with human papillomavirus serotype 16 [8]. IBC-10a [9] cells were primary 'intermediate basal cell' cultures derived from a Gleason score 6 prostate cancers by our lab. IBC-10a cells were subsequently immortalized with hTERT (courtesy of Johng Rhim, Bethesda, MD). The IBC-10a cells were also transfected with a pBABE-c-myc puromycin vector (courtesy of Dr. Sell, Drexel Univ., Philadelphia, PA)(the pBABE vector was purchased from Clonetics Inc., Boston, MA)) and stable clones selected for 2 weeks with 2 *ug/ml *puromycin. The CPTX-1532 and NPTX-1532, BPH-1, and IBC-10a were maintained at low passage (< 10) in Keratinocyte serum free media (SFM) (Life Technologies, Inc., Grand Island, NY) containing 5 *ng/mL *epidermal growth factor, 50 *μg/mL *bovine pituitary extract, plus 100 units/mL penicillin G sodium and 100 *μg/mL *streptomycin sulfate. Cells were cultured at 37°C in a humidified atmosphere of 95% air and 5% CO_2_. In experimental studies, the cells were harvested using a 1:10 dilution of 10X Trypsin-EDTA (Invitrogen, Grand Island, N.Y.) in PBS, pH 7.4 and washed three times with fresh keratinocyte SFM, counted with a hemacytometer, and plated in keratinocyte SFM o.n. prior to starting experimental treatments. Cell growth assays were carried out using MTS reagents according to methods of the manufacturer (Promega Inc., Madison, WI).

### Recombinant RPS2 protein

The RPS2 cDNA isolated from PC-3ML cells was inserted into a phagemid ZAP expression vector system using a protocol described by the manufacturer (Stratagene Inc., La Jolla, CA). A pGEXR-GST fusion protein was cloned in BL21 (DES) pLysS E. coli. The cDNAs from 3 clones was sequenced by the DNA facility (Univ. of Pennsylvania) to verify the gene. Recombinant GST-RPS2 protein was purified using the MagneGST protein purification system according to a protocol provided by the manufacturer (Promega Inc.).

### PCR primers for RPS2

Total RNA (1 *μg*) was reverse transcribed using the SUPERSCRIPT™ II Rnase H^- ^Reverse Transcriptase System. Samples were subjected to PCR amplification in a total reaction volume of 50 μl containing 10× PCR buffer (GIBCO BRL^®^), 50 *mM *MgCl_2 _(GIBCO BRL^®^), 10 *mM *dNTP, 5 pmol concentration of each specific primer, and 2.5 units of *Taq *DNA polymerase (GIBCO BRL^®^). The PCR reaction was carried out in a programmable thermal controller (PTC-100, MJ Research, Inc., Watertown, MA). The reaction mixture was denatured at 94°C for 3 min followed by 30 cycles at 94°C for 45 s, annealing at 60°C for 45 s and 72°C for 1 min. The final elongation was extended for an additional 20 min. The amplified PCR products were resolved electrophoretically on agarose gel stained with ethidium bromide to verify size of the amplified product [10]. Also, the identity of RPS2 fragments was verified by nucleotide sequencing (Molecular Sequencing Facility, Univ. Pennsylvania, Philadelphia, PA).

Forward Primer: 5': GCCAAGCTCTCCATCGTC-3' 18 MER, TM: 59.8

Reverse Primer: 5'-GTGCAGGGATGAGGCGTA-3': 18 MER, TM: 60.6

Melting curve analyses showed a clean primer dimer free RPS2 DNA peak (90°C). PCR reactions were repeated twice to confirm the size of the 350b products (30 cycles) seen on the agarose gels [10]. The Stratagene cDNA was used as a positive control.

### DNAZYM-1P (31b)

**Figure 1:**
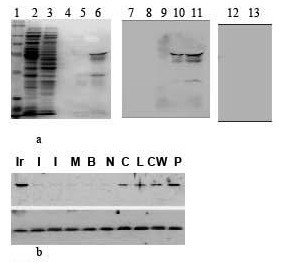
**a (Lanes 1–6) SDS PAGE of (lane 1) mwt markers; (lane 2) crude bacterial cell lysate containing the GST-RPS2 fusion protein; (lanes 3–4) unbound proteins; (lanes 5–6) GST-RPS2 fusion protein bound to the MagneGST Glutathione Particles; (lanes 7–11) RPS2 antibody (1:1000 dil.) Western blots of proteins in lane 2, 3, 4, 5, and 6, respectively**. (lanes 12–13) Western blots of fractions in lanes 5–6 following preabsorption of the P1 antibodies (1:200 dil.) with excess recombinant RPS2 (200 *ng*). Note: the P1 antibodies blotted 2 different bands of the GST-RPS2 complex at ~62 Kda plus the 33 Kda RPS2 protein. **1b**. Western blots with RPS2 antibodies (1:1000 dil.) of nuclear protein extracts from: (Ir) pBABE-IBC-10a-c-myc; (I) 2 different IBC-10a sub-clones; (M) mouse NIH-3T3; (B) BPH-1, (N) NPTX-1532, (C) CPTX-1532, (L) LNCaP, (CW) CRW22R1, and (P) PC-3ML cells. Lower bands: actin antibody blots of nuclear extracts. Loaded at 20 ug/lane.

The DNAZYM-1P was designed with two flanking 8 base sequences which recognize the RPS2 mRNA and a 15 base catalytic domain known as the '10–23' motif as the core. The DNAZYM-1P was similar in design to the DNZYM previously developed by others for targeting HIV-1 gag, c-myc, and egr-1 RNA, respectively [11-14]. (fig. 1S, additional file 1). A 'scrambled' DNAZYM was made with random flanking sequences and the 15 base catalytic domain (fig. 1S). The sequences for 2 different DNAZYMs are shown below and include the flanking regions (8 bases) and catalytic domain (underlined). Note: Both DNAZYM-1P and 2P exhibited similar potency and only the data from the DNAZYM-1P is reported in this paper. The DNAZYMs were synthesized and purified by BioSource International (Camarillo, CA)..

DNAZYM-1P: GATCTTCAGGCTAGCTACAACGAGTCCTTGA

DNAZYM-2P: GTTCCCCAGGCTAGCTACAACGACCCAGGGC

### SCID mouse tumor modeling studies

The studies were carried out utilizing 6–8 week old male CB17-SCID mice (Severe Combined Immunodeficient Mice, Taconic Labs, Germantown, N.Y.) according to previously published methods [15]. PC-3 ML tumor cells were derived from parent PC-3 cells after repeated selection of the invasive PC-3 cells utilizing Matrigel coated modified Boyden Invasion Chambers [5] (BD Biosciences, Franklin Lakes, N.J.). Invasive cells were then injected i.v. in SCID male mice and single cell clones isolated from the bone marrow tumors [5]. Two types of studies were carried out. First the PC-3ML cells were inoculated s.c. in the scrotal pouch (0.2 ml at 5 × 10^6 ^cells) prior to initiation of treatment on day 28. Mice were then treated by localized injection of the DNAZYM-1P (4.0 *ug*/1 *ml *in 0.1 *ml *biw). Secondly, cells were injected i.v. via the tail vein (0.2 *ml *at 1 × 10^5 ^cells) twice at 10 day intervals, and once tumors were established, treatment was initiated day 20. Mice were then treated by i.v. injection via the tail vein of the DNAZYM-1P (i.e. 4.0 *ug*/*ml *in 0.1 *ml *weekly). In controls, mice were injected with the scrambled DNAZYM or lipofectamine 2000 (vehicle) (Invitrogen). Immediately prior to injection, the DNAZYM-1P resuspended in DMEM was incubated with 20 *uM *lipofectamine 2000 for 1 hr at room temperature.

### Western blots and immunolabeling

SDS PAGE, Western blots and protein measurements were carried out according to methods previously described by out lab [5,10,15].

## Results

### PCR analysis

**Figure 2:**
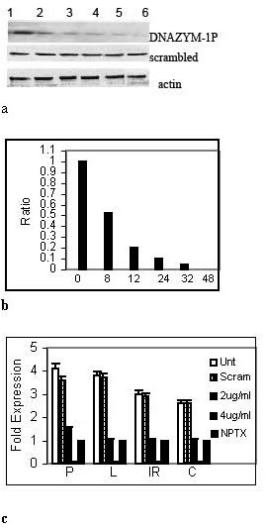
**a Western blots with RPS2 antibodies of crude cell extracts from PC-3ML cells following treatment with (top lane) 20 *nM *DNAZYM-1P for increased times of (lanes 1–6) 0, 8, 12, 24, 32 and 48 hr, respectively**. Control cells were treated for identical times with (middle lane) 20 nM scrambled oligonucleotide. (bottom lane) beta actin antibody blots. **2b**. Comparisons of the ratio of RPS2/actin from densitometry scans of the Western blots in fig. 2a. **2c**. RT-PCR assays showing the relative level of RPS2 expression in (P) PC-3ML; (L) LNCaP; (IR) pBABE-IBC-10a-c-myc; and (C) CPTX-1532 cells (at 90% confluent) which were (□) untreated or treated with (╪) scrambled oligonucleotide, and (░) 2 and (■) 4 *ug/ml *DNAZYM-1P for 8 hr. Shows that the DNAZYM-1P knocks out RPS2 mRNA expression in all 4 cell lines. (×) NPTX-1532 express low levels of RPS2 mRNA (value set at 1). RT-PCR vales were normalized relative to 18S RNA, and then the fold expression calculated relative to values for untreated NPTX-1532 cells which were set at 1. Results averaged from 3 experiments +/-1 S.D.

PCR primers specific for the n-terminal domain of the RPS2 mRNA revealed that 3 different malignant PCa cell lines (i.e. LNCaP, PC3-ML, DU145) and 3 pre-malignant or partially malignant lines (HGPIN, CPTX-1532, pBABE-IBC-10a-cmyc) over expressed the RPS2 mRNA. The mRNA (i.e. cDNA after 35 cycles) was barely detectable in several non-malignant primary cell strains, including BPH-1, IBC-10a and NPTX-1532 cells, and was not present in 3T3 fibroblasts (fig. 2S, additional file 1). Sequencing of the 350b fragments revealed a 100% homology with the RPS2 mRNA.

### Western blot studies

Crude protein extracts (100 *mg/ml*) from BL21 *E. coli *containing recombinant pGEXR-GST-RPS2 fusion protein were incubated with MagneGST Glutathione Particles and the magnetic beads removed with a magnet. Following three washes with the binding buffer to remove unbound protein (fig. 1a, lanes 3–4), GST-RPS2 fusion protein was recovered by elution with 50 mM glutathione (fig. 1a, lanes 5–6). Western blots with RPS2 antibodies revealed that the ~62 Kda GST-RPS2 complex contained RPS2 (fig. 1a, lanes 10–11). A lower molecular weight band at 33 Kda was also blotted with the RPS2 antibodies (fig. 1a, lanes 10–11). Control blots with RPS2 antibody pre-absorbed with purified rRPS2 protein, failed to blot the GST-RPS2 protein complex (fig. 1a, lanes 12–16). Blots with GST antibodies (1:400 dil.) blotted only the 62 Kda of GST-RPS2 protein complex (not shown). Western blots of nuclear protein extracts from human prostate cell lines showed that RPS2 was abundantly expressed in several malignant prostate cancer cell lines, including: pBABE-IBC-10a-c-myc (Ir), CPTX-1532 (C), LNCaP(L), CRW22R1 (CW), and PC-3ML (P) cells, but was not expressed (or faintly expressed) in normal prostate cell lines, including two different sub-clones of parent IBC-10a cells (I), mouse NIH 3T3 fibroblasts, BPH-1, and NPTX-1532 cells (fig. 1b).

### DNAZYM-1P studies

Western blots showed that a DNAZYM-1P designed to target the n-terminal ATG start site of the RPS2 mRNA protein 'knocked-down' the detectable levels of nuclear RPS2 protein in PC-3ML cells after 8–48 hr treatment (fig. 2a, top lane). Controls showed that a DNAZYM-1 with scrambled base sequences in the flanking regions of the DNAZYM (i.e. scrambled oligonucleotide) failed to 'knock-down' RPS2 expression after 0–48 hr (fig. 2a, middle lane). Densitometry scans of the bands and comparisons of the ratio of RPS2/actin showed that the relative level of RPS2 expression dropped from 1 to 0.5, 0.2, 0.1, 0.05 and < 0.02 following treatment of the PC-3ML cultures with DNAZYM-1P for 0, 8, 12, 24 32 and 48 hr, respectively (fig. 2b). RT-PCR assays with primers specific for RPS2 confirmed that the 2 and 4 *ug/ml *DNAZYM-1P 'knocked-down' expression of RPS2 mRNA after 8 hr in PC-3ML (P), LNCaP (L), pBABE-IBC-10a-c-myc (IR) and CRW22R1 (C) cells. The fold expression of RPS2 mRNA in the 4 different cell lines was normalized to 18S RNA and then the fold expression calculated relative to RPS2 mRNA levels in untreated NPTX-1532 cells (value set at 1) (fig. 2c). The scrambled oligonucleotide failed to significantly alter RPS2 mRNA levels in any of the cell lines, however (fig. 2c).

**Figure 3:**
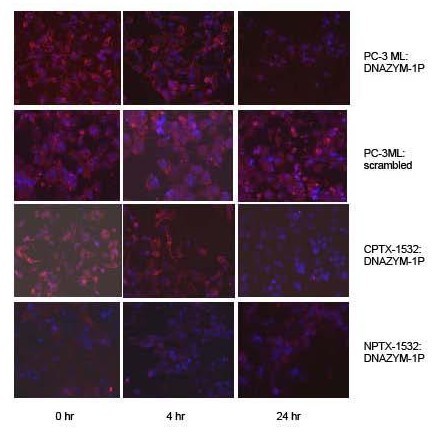
**Immunolabeling of PC-3ML, CPTX-1532 and NPTX-1532 cells with RPS2 antibodies following treatment with 4 *ug/ml *DNAZYM-1P or scrambled oligonucleotide for 0, 4 and 24 hr**. Cells were labeled with RPS2 P1 antibody (1:200 dil.) and Alexoflour secondary antibodies counterstained with DAPI. Cells were at ~70% confluence at the time treatment was initiated.

Immunoflourescent labeling studies with RPS2 antibodies (i.e. P1 antibodies) revealed that RPS2 was over expressed in nuclear and cytoplasmic regions of untreated PC-3ML and CPTX-1532 cells (fig. 3). Figure 3 showed that following exposure of these cells to DNAZYM-1P (4 *ug/ml*) for 0 and 4 hr, the cells expressed an abundance of RPS2 (fig. 3). However, following extended treatments of 24 hr, the majority of the cells were negative for RPS2. Control experiments showed that PC-3ML cells exposed to the scrambled DNAZYM oligonucleotide expressed RPS2 after 0, 4 and 24 hr. In comparison, NPTX-1532 cells which did not express RPS2, were unaffected by DNAZYM-1P for 0, 4 and 24 hr (fig. 3). IBC-10a parent cells also did not express RPS2 or respond to DNAZYM-1P treatment (data not shown).

**Figure 4:**
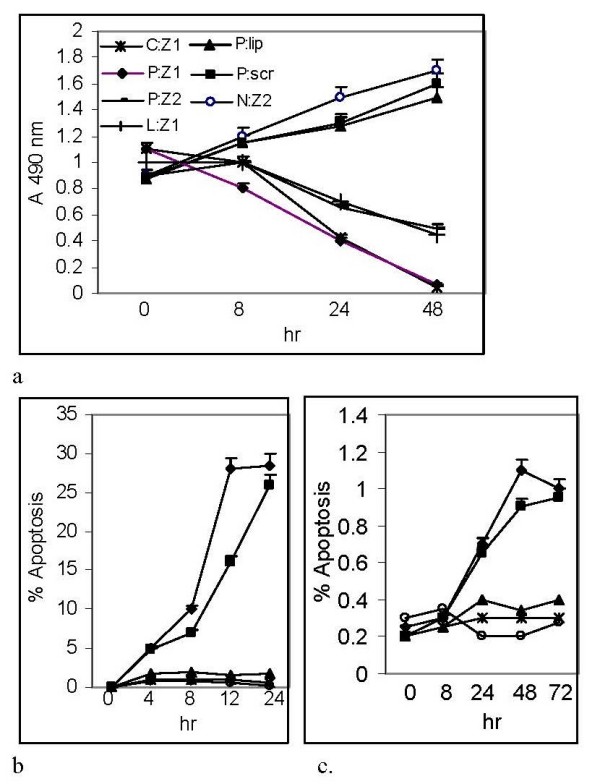
**a MTS assays showing that 4 or 6 *ug/ml *DNAZYM-1P (i.e. Z1 and Z2, respectively) treatment of 90% confluent cultures not only blocked cell growth, but reduced the cell density after 8, 24 and 48 hr, respectively, in (P:Z1, P:Z2) PC-3ML, (L:Z1) LNCaP, and (C:Z1) CPTX-1532 cells**. The growth of (N:Z2) NPTX-1532 cells was not blocked by 6 *ug/ml *DNAZYM-1P treatment after 0, 8, 24 and 48 hr, however. Controls showed that growth of PC-3ML cells treated with lipofectamine (P:lip) or a 6 *ug/ml *scrambled DNAZYM oligonucleotide (P:scr) was not blocked. **4b-4c**. Apoptosis Assays using annexin V antibody labeling and flow cytometry. Showed that 4 & 6 *ug/ml *DNAZYM-1P (■, ◆) induced increased amounts of apoptosis in (fig. 4b) PC-3 ML cells after 8–24 hr (i.e. 5% to 28%), but failed to induce apoptosis in (fig. 4c) NPTX-1532 cells after 0, 8, 24, 48 and 72 hr treatment (i.e. < 1.2%). Controls showed that (▲) lipofectamine, (○) scrambled DNAZYM oligonucleotide, or (Ж) untreated cells exhibited very low levels of apoptosis.

Growth assays measured by the MTS assay [8] further showed that 4 and 6 *ug/ml *DNAZYM-1P blocked growth of 3 different malignant prostate cancer lines which over expressed RPS2, including PC-3ML (P:Z1, P:Z2), CPTX-1532 (C:Z1) and LNCaP (L:Z1) cells. In comparison, the scrambled oligonucleotide (P:scr) and lipofectamine (P:lip) alone did not block growth of PC-3ML cells. DNAZYM-1P treatment of NPTX-1532 (N:Z2) cells did not block cell proliferation (fig. 4a). Apoptosis Assays using Annexin V antibody labeling and flow cytometry showed that 4 & 6 *ug/ml *DNAZYM-1P induced increased amounts of apoptosis in PC-3ML cells after 8–24 hr (i.e. 5% to 28%) (fig. 4b, ■, ◆), but failed to induce significant amounts of apoptosis in NPTX-1532 cells after 0, 8, 24, 48 and 72 hr treatment (i.e. < 1.2%)(fig. 4c, ■, ◆). Control studies confirmed that lipofectamine (▲) alone or the scrambled DNAZYM oligonucleotide (○), and vehicle (Ж) failed to induce apoptosis in either PC-3ML (fig. 4b) or NPTX-1532 (fig. 4c) cells. In other studies, pBABE-IBC-10a:c-myc cells which over expressed RPS2 exhibited high levels of apoptosis of 9% and 30% by 8 and 24 hr in response to 6 *ug/ml *DNAZYM-1P (data not shown).

### SCID mice tumor modeling studies

**Figure 5:**
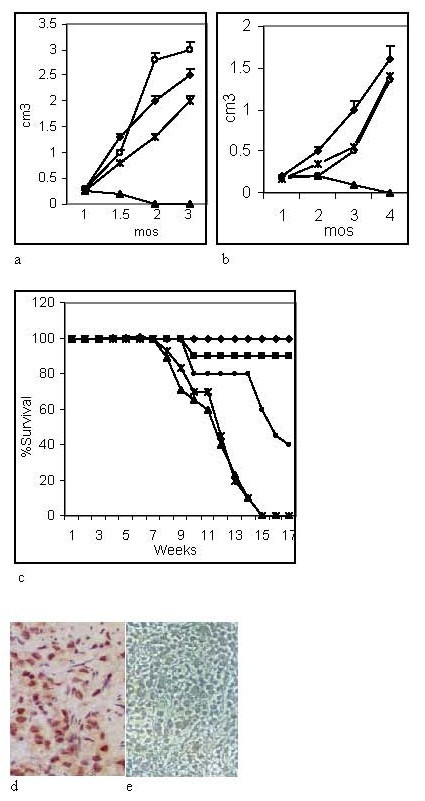
**a Mice were injected in the scrotal sac with 1 × 10^6 ^PC-3ML cells**. Treatment was initiated at day 28, and mice treated with (▲) 4 *ug/biw *DNAZYM-1P) (n = 50); (◆) scrambled oligonucleotide (n = 30); (Ж) vehicle (n = 20) or (○) untreated. The agent was injected in the scotal sac in 0.1 *ml *buffer. Tumor size was measured with calipers at 2 week intervals. **5b**. Mice (n = 30/agent) were injected i.v. via the tail vein at day 1 and day 10 with 1 × 10^5 ^cells/ml (in 0.1 ml) then treatment started after 2 weeks by i.v. injection via the tail vein weekly of (◆) scrambled oligonucleotide, (○) vehicle, (Ж) buffer, and (▲) DNAZYM-1P. Tumor volume was measured in 2 mice at 2 weeks by sacrificing a few mice for measurements and then at the time of sacrifice following treatment of mice for 1, 2, 3 and 4 mos. **5c**. Mice were injected with tumor cells according to methods in fig. 5b and treated with either (◆) 4 *ug/ml*, (■) 3 *ug/ml *and (●) 2 *ug/ml biw DNAZYM-1P. Control mice were treated with *(▲) lipofectamine and (Ж) scrambled oligonucleotide. Mice were treated for 2 mos, then treatment was discontinued for up to 17 weeks. **5d–5e**. H&E and RPS2 antibody immunolabeled sections of a tumor from a mouse treated with the scrambled oligonucleotide for 2 mos (see fig. 5c).

Tumor modeling studies were carried where PC-3ML tumor cells were injected in the scotal sac of 8 week old SCID mice. Since the testis do not descend by 8–14 weeks of age, it was possible to inject in the scotal sac where the bulk of the cells or reagent tend to remain following injection. We allowed the tumors to establish and reach a size that was palpable after 28 days prior to initiating treatment with the DNAZYM-1P. Mice were then treated for ~2 mos at a dosage of 4 *ug/biw injected topically in the scrotal sac*. In mice treated with 4 *ug/ml *biw DNAZYM-1P (▲)(n = 50), 33/50 mice exhibited no detectable tumors and 12/50 had tiny nodules (< 0.2 cm^3^) which were hollow spheres coated by collagen networks and empty of tumor cells. In untreated mice (○) (n = 20) or mice treated with the scrambled oligonucleotide (◆)(n = 30) or vehicle (n = 20) (Ж) the tumors reached a size of 2–2.6 cm^3 ^after ~2 mos and all the mice had scrotal sac tumors plus localized metastases to the peritoneal cavity (fig. 5a). None of the mice exhibited detectable metastases (fig. 5a).

Similar studies were then carried out to assess whether DNAZYM-1P delivered systemically, could block the growth of tumors disseminated to a variety of organ systems. In these experiments, mice were injected i.v. via the tail vein at day 1 and day 10 with 1 × 10^5 ^cells/ml then treatment started after 2 weeks by i.v. injection via the tail vein of DNAZYM-1P (▲)(n = 30), scrambled oligonucleotide (◆)(n = 30), vehicle (○)(n = 30), or buffer (Ж)(n = 30). The data in fig. 5b showed that tumors did not survive in mice treated with DNAZYM-1P (▲), whereas numerous tumors were found in the kidney, sternum, peritoneum, liver and lungs of mice treated with scrambled oligonucleotide (◆), vehicle (○) or buffer (Ж).

Mouse survival studies were then carried out under the conditions described in fig. 5b, where treatment with the different agents was discontinued after 2 mos and the mice monitored for ~4 mos. The mouse survival data showed that the mice all died by ~7–15 weeks in mice treated with lipofectamine (▲) or scrambled oligonucleotide (Ж) (fig. 5c). In mice treated with 2, 3 and 4 *ug/ml *DNAZYM-1P, mouse survival was either (●) 40%, (■) 90% and (◆) 100%, respectively. H&E stained sections and RPS2 antibody labeled sections of the tiny tumors present at the time treatment was initiated, showed that the PC-3ML cells normally formed solid tumor masses and the cells over expressed RPS2. In mice treated with the scrambled oligonucleotide for 2–3 mos, the tumors still consisted of a packed mass of PC-3ML cells (fig. 5d) which expressed RPS2 (fig. 5e). Residual nodules sometimes remained following treatment of the mice with DNAZYM-1P for 2 mos. These nodules consisted of a collagen shell, but were largely empty masses filled with debris that was not immunolabeled with RPS2 antibodies (data not shown). Overall, we found that DNAZYM-1P treatment of the mice appeared to be of low or zero toxicity to the mice since they gained weight on a regular basis, were robust and healthy in appearance and showed zero neuropathy or hair loss. Histology of the liver, kidney, spleen, brain, spine, lungs, and heart indicated normal undamaged tissue. Also the growth of fibroblast cultures from the prostate tissue of mice was not hindered by 4 and 6 *ug/ml *DNAZYM-1P, indicating the DNAZYM-1P probably does not target cells which express low levels of RPS2 protein.

## Discussion

Earlier immunolabeling studies with polyclonal antibodies had revealed that the RPS2 antigen was over-expressed in 100% of prostate cancer luminal epithelial cells (n = 20 prostates examined). In contrast, the protein was not expressed in NPTX-1532, benign prostate hyperplasia (BPH), seminal vesicle (SV) or in skeletal or smooth muscle tissues from the same prostates with (or without) cancer foci [1]. Likewise, RPS2 (aka: PCADM-1) was not expressed by primary prostate tissue fibroblast cultures, WI38 human fibroblasts, human peripheral blood lymphocytes or human hepatocyte cultures [1].

In this paper, we have examined whether the PCADM-1 gene/protein is normally over expressed in malignant prostate cancer. Western blots indicated benign prostate did not express the protein, whereas malignant prostate cancer expressed PCADM-1 and the amount of RPS2 expressed increased with the tumor grade. We have, therefore, focused on studies designed to test whether RPS2 over expression in prostate cancer cell lines is essential for cell survival. To our surprise, we found in 'anti-sense' knock-out experiments with a DNAZYM-1P which targeted the RPS2 mRNA, that gene expression was essential for cell survival, but only in cells which over expressed the RPS2 protein (i.e. in PC-3 ML, LNCaP, CPTX-1532 and pBABE-IBC-10a-c-myc cells). In comparison, prostate cell lines expressing very little RPS2 (i.e. BPH-1, NPTX-1532 or IBC-10a cells) were not affected by the DNAZYM-1P treatment even at high concentrations for prolonged intervals. That is, only the PC-3ML and pBABE- IBC-10a-c-myc cells which expressed elevated RPS2 underwent apoptosis and failed to grow in response to DNAZYM-1P. NPTX-1532 or IBC-10a cells which failed to express detectable RPS2 did not undergo apoptosis. Likewise, DNAZYM-1P treatment of localized or metastatic tumors in SCID mice, completely eradicated the tumors, but did not inflict noticeable harm to normal mouse cells. We interpret this to mean that the over-expression of RPS2 might promote ribosomal biogenesis and growth of tumor cells and that the tumor cells acquire a dependence on RPS2 for survival. Thus, 'knock-out' of RPS2 results in a 'shut-down' of ribosomal biogenesis and a cascade of apoptotic events leading to inhibition of cell growth and apoptosis. Again, a similar response was not observed in normal cells since the temporary 'knock-down' of RPS2 mRNA had little impact on overall cell homeostasis.

Perhaps more importantly, we found that DNAZYM-1P treatment of tumor bearing mice was a highly effective therapeutic approach to eradicating tumors and dramatically improving disease free mouse survival rates. We showed that the DNAZYM-1P eliminated PC-3ML tumors in mice (> 90%) and that treatment resulted in a significant increase in disease free mouse survival rates (> 80–100%) after discontinuation of the treatment for ~4 mos. The DNAZYM-1P was non-toxic after prolonged treatment intervals and following the discontinuation of treatment, tumor recurrence was not detected in the liver, lungs, peritoneum or other organs after several months indicating the mice were cured of cancer. The implication is that targeting RPS2 in prostate cancer might be an excellent therapeutic strategy.

A number of studies have previously shown that the over expression of different ribosomal proteins might play an important role in cancer. Chiao et al. [16] has shown that RPS2 ribosomal mRNA was over expressed in head and neck cancer and barely detectable in normal tissue. Others have found that the rat ribosomal protein S3a is identical to rat v-fos transformation effector protein [17]. Karan et al. [18] found 34 genes are up-regulated and eight genes are down-regulated in androgen-independent prostate cancer cells, including L10 (RPL10), L32 (RPL32), and S16 (RPS16). It therefore appears that independent, non-coordinate changes in expression of a subset of ribosomal proteins, might occur which have no direct association or correlation with proliferative and/or protein synthetic activities involved in ribosomal biogenesis [4,19,20], but could be involved in transformation [21,22]. For example, studies by Naora et al. [22] showed that enhancement of RPS3a expression in NIH 3T3 cells induced transformation and formation of tumors in nude mice and they found that S3a expression was a critical gene for tumor cell survival and tumorigenesis.

Like S3a, our data suggested that over expression of RPS2 was associated with prostate tumor formation and key for tumor cell survival. The interesting aspect of these studies is that suppression of enhanced RPS3a or RPS2 expression both could be associated with and/or involved in a downstream pathway which leads to apoptosis. For example, S-12 cells that over express RPS3a, undergo apoptosis when enhanced RPS3a expression was inhibited [22].

There is some precedent for this suggestion. There are cases where growth inhibition and/or apoptosis have been induced by switching off expression of c-*myc *and *bcr-abl *in promyelocytic, and in chronic myeloid, leukemia cells, respectively [23,24]. Thus, it is possible that apoptotic induction might arise as a default event when RPS3a or RPS2 expression is blocked, simply from an inadvertent inhibition of survival factors. Unfortunately, the physiological signals that mediate such suppression are probably cell specific and obviously remain to be elucidated.

As pointed out in the introduction, there are many reports showing a connection between over-expression of genes encoding ribosomal proteins and cancer [16,17,25-32]. The implication is that these ribosomal proteins have additional functions distinct from their role as ribosomal proteins regulating protein synthesis [16,17,25-32]. In this respect, specific 'leucine zipper' sequence motifs are characteristic of numerous ribosomal proteins which allow binding to nucleic acids [16,17,26,29-31] and a possible role in regulating transcriptional and translational mechanisms. For example, the rat ribosomal protein S3a is identical to the product of the rat v-fos transformation effector gene [29]. And S3a is normally involved in initiation of protein synthesis and is related to proteins involved in the regulation of growth and the cell cycle [4]. In one study, over expression of S3a was able to induce transformation of NIH 3T3 cells and induce formation of tumors in nude mice [33]. But the ability of S3a to induce transformation was dependent on its role in suppressing programmed cell death [33]. A second example is the rat ribosomal protein L10. L10 is homologous to a DNA-binding protein and to a putative Wilm's tumor suppressor gene [28]. A third example is S19 where a mutation in the S19 ribosomal protein has been associated with a predisposition to cancer in patients with Diamond-Blackfan anaemia [34]. Finally, RPS2 was shown by our lab to specifically bind a classical 'break point cluster region' sequence found in leukemia [35], implicating RPS2 as a DNA binding protein. The DNA binding domain is a leucine zipper domain where 4 point mutations have been detected. Thus, aberrant over expression of RPS2 or the mutant form of RPS2 (termed PCADM-1) might somehow activate oncogenes involved in tumor development. In this connection, the individual and/or combined effects of a variety of ribosomal proteins (i.e. like RPS2, S3a, L10, and L19) might directly control gene expression patterns, oncogene expression and transformation.

## Conclusion

We believe that targeting one or more of these ribosomal proteins (i.e. RPS2 or S3a) may lead to development of a highly effective treatment for prevention of cancer, eradication or primary tumors or a blockade of tumor metastasis.

## Abbreviations

RPS2, RPS3, S5, RPS6, RPS8, RPS12 and S14: Ribosomal protein S2, S3a, S5, S6, S12 and S14, respectively; L5, L7, L10, L16, L19 and L37: Ribosomal protein L5, L7, L10, L16, L19 and L37; HGPIN: High grade prostatic neoplasia; BPH: Benign prostatic hypoplasia; SCID mice: Severe Combined Immunodeficient mice; SV: Seminal vesicle.

## Competing interests

The authors declare that they have no competing interests.

## Authors' contributions

All authors contributed equally to the research work and writing of the manuscript.

## Supplementary Material

Additional file 1 **Illustrates the basic design of the DNAZYM-1P construct. **Shows 8b flanking regions which correspond to specific sequences in the 5' region of the RPS2 mRNA. The 15 b core of the DNAZYM-1P constitutes the catalytic domain, the '10-23' motif [11]. Click here for file
