# Supplementary figures and images for: RPS2: a novel therapeutic target in prostate cancer
Source: J Exp Clin Cancer Res. 2009 Jan 12;28(1):6. doi: 10.1186/1756-9966-28-6 (PMC2633276; doi:10.1186/1756-9966-28-6)

mRNA  
DNAZYME

5'-UCAAGGACA\*  
3'-AGTTCCTG

UGAAGAUC  
ACTTCTAG

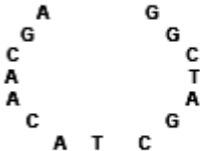

Supplement: Additional file 1 — Illustrates the basic design of the DNAZYM-1P construct. Shows 8b flanking regions which correspond to specific sequences in the 5' region of the RPS2 mRNA. The 15 b core of the DNAZYM-1P constitutes the catalytic domain, the '10-23' motif [11]. [file 1756-9966-28-6-S1.pdf]
